# Effect of pre-hospital living setting on nutritional intake route upon discharge in older adults with aspiration pneumonia: a prospective cohort study

**DOI:** 10.1186/s12877-024-05659-x

**Published:** 2025-01-04

**Authors:** Kohei Yamaguchi, Taiju Miyagami, Ryoko Imada, Ryosuke Yanagida, Seiko Kushiro, Toru Morikawa, Kazuharu Nakagawa, Kanako Yoshimi, Toshio Naito, Haruka Tohara

**Affiliations:** 1 Department of Dysphagia Rehabilitation, Institute of Science Tokyo, 1-5-45 Yushima, Bunkyo-ku, Tokyo, 113-8510 Japan; 2 Department of General Medicine, Juntendo University Faculty of Medicine, Hongo 2-1-1, Bunkyo-ku, Tokyo, 113-8421 Japan; 3 Department of General Medicine, Nara City Hospital, 1-50-1 Tokijicho, Nara City, Nara 630-8305 Japan

**Keywords:** Pneumonia, Deglutition disorders, Nursing facility, Multidisciplinary research, Health care survey, Aging

## Abstract

**Background:**

Aspiration pneumonia, which often recurs due to dysphagia, worsens as patients move between homes, facilities, and hospitals. The impact of pre-hospital living setting on oral intake at discharge remains unclear. The purpose of this study was to identify the effects of the pre-hospital living setting on the nutritional intake route upon discharge in older patients with aspiration pneumonia.

**Methods:**

This prospective cohort study included patients aged ≥ 65 years who were admitted to an acute care hospital and diagnosed with aspiration pneumonia. Patients were followed up until discharge or death during hospitalisation. Patient demographic information, pre-hospital living setting (home or nursing facility), functional oral intake scale (FOIS) score, pneumonia severity index, clinical frailty scale score, history of aspiration pneumonia, and pneumonia recurrence during hospitalisation were recorded. Binary logistic regression was used to assess the impact of the pre-hospital living setting on oral intake at discharge as the primary outcome.

**Results:**

Among the 89 included patients (52 males (58.4%); mean age, 84.8 ± 7.9 years), 39.3% (*n* = 35) had pneumonia recurrence during hospitalisation. The average follow-up period was 44.0 ± 36.6 days. The pre-hospital living setting was independently associated with the nutritional intake route upon discharge (odds ratio = 7.72, 95% confidence interval (95%CI) = 1.70–35.1, *p* = 0.008).

**Conclusions:**

The pre-hospital living setting could serve as a good indicator of the nutritional intake route upon discharge. It is essential to optimize care in both nursing facilities and hospital settings when caring for older patients with aspiration pneumonia.

## Key summary points

• **Aim** To identify the effect of pre-hospital living setting on nutritional intake route upon discharge in older adults with aspiration pneumonia.
• **Findings** The pre-hospital living setting significantly affected the nutritional intake route upon discharge, as did pneumonia recurrence during hospitalisation and frailty.
• **Message** When caring for older patients with aspiration pneumonia, attention should be paid to both their physical condition and pre-hospital living setting, as these are useful predictors of the nutritional pathway at discharge.

## Introduction

The global population aged > 65 years is expected to double between 2019 and 2050 [1]. Aspiration pneumonia is commonly observed in older patients with pneumonia, with a prevalence estimated to be as high as 80% (306/382) of all cases [2]. In a survey of hospitalised patients, aspiration pneumonia was reported in 60.1% (264/439) of patients with community-acquired pneumonia (CAP) and 86.7% (130/150) of those with hospital-acquired pneumonia (HAP) [2]. Recurrence is a typical characteristic of aspiration pneumonia both in hospitals and in the community. The recurrence rate within 1 month of hospitalisation was 32.4% (47/145) [3]. A survey of the causes of re-hospitalisation in patients with CAP revealed that 39.6% (516/1304) were related to pneumonia [4].

Aspiration pneumonia is caused by aspiration of oral bacteria, and dysphagia is a major related risk factor [5]. Dysphagia reportedly increases the risk of developing pneumonia nine-fold in frail community dwelling older adults [6]. Dysphagia occurs at a high rate in older patients with pneumonia in acute care hospitals [7]. The nutritional pathway at discharge, such as tube feeding or parenteral nutrition, is a major prognostic factor for patients with aspiration pneumonia [8]. On the other hand, in community settings, there can be a mismatch between swallowing ability and diet texture, meaning some individuals may eat food with textures not suited to their swallowing function [9].

The concept of nursing and healthcare-associated pneumonia (NHCAP) has been introduced, positioned between CAP and HAP, specifically targeting older individuals receiving care in long-term care hospitals and nursing facilities. The prevalence of frailty in nursing facilities has been reported to range from 28.5 to 70% [10], which is higher than the prevalence reported in community-based surveys [11]. A cohort study in the United Kingdom has also found that poor prognostic factors in older patients with pneumonia depend more on frailty and overall condition than on whether the pneumonia is aspiration-related [12]. Almost 40% of older adults receive care in facilities, reflecting the increasing diversity in the living environments of older adults [13]. Considering the recurrent nature of aspiration pneumonia, the condition of patients with aspiration pneumonia worsens as they are admitted and discharged. In this case, the pre-hospital living setting and discharge destination (pre-hospital living setting in the case of re-admission) can be clinically important predictors for patients with aspiration pneumonia. Dysphagia and nutritional intake routes are also factors that determine the discharge destination [14], contributing to a vicious circle [15].

However, the effect of the pre-hospital living setting (home or nursing facility) on the nutritional intake route upon discharge of older patients with aspiration pneumonia remains unclear. Therefore, the primary aim of this study was to identify the effect of pre-hospital living setting on oral intake upon hospital discharge in older patients with aspiration pneumonia. The secondary aims were to investigate the effect of pre-hospital living setting on the presence of dysphagia at admission and to examine changes in nutritional intake route during hospitalisation. We hypothesised that older patients with aspiration pneumonia who were admitted from nursing facilities would be more likely to require tube feeding upon discharge.

## Methods

### Research design and settings

This prospective cohort study was conducted at Juntendo Tokyo Koto Geriatric Medical Centre between April 2021 and March 2022. Juntendo Tokyo Koto Geriatric Medical Centre is an acute care hospital with 404 beds that provides secondary emergency care specifically for older adults. The hospital houses a total of 24 medical departments, including a department of general medicine, with 682 staff members, including specialists in various fields. The average number of inpatients per day is 368. The hospital serves as a regional hub for the management of acute illnesses in older populations.

### Participants

#### Inclusion criteria

The participants in this study were patients aged 65 years or older who were admitted to Juntendo Tokyo Koto Geriatric Medical Centre with a diagnosis of aspiration pneumonia between April 2021 and March 2022. During this period, the patients admitted for aspiration pneumonia came from two kinds of pre-hospital setting, namely private homes and nursing facilities. All were admitted to the hospital following transport by ambulance or through a referral due to the severity of their condition.

The diagnosis of aspiration pneumonia for admitted patients was made by doctors at the Juntendo Tokyo Koto Geriatric Medical Centre. The doctors who primarily performed the diagnoses and treatments in this study had more than 10 years of experience. The diagnosis of aspiration pneumonia was based on (1) chest radiography and computed tomography images consistent with aspiration pneumonia; (2) aspiration witnessed by family or caregivers (such as a coughing episode with food or an asphyxiation incident), or a risk for aspiration pneumonia; and (3) acute symptomatic pneumonia (fever, cough, phlegm [15]). Patients who met all the criteria were diagnosed with aspiration pneumonia.

#### Exclusion criteria

Exclusion criteria included missing data and diagnoses of lung cancer, tuberculosis, and viral pneumonia for the study to specifically focus on cases of bacterial pneumonia caused by aspiration.

### Measurement of oral intake

The functional oral intake scale (FOIS) score was used to assess oral intake [16, 17]. The scale scores range from 1 (no oral intake and alternative nutrition only, including nasogastric tube feeding or peripheral intravenous therapy) to 7 (no restrictions on oral intake), with scores of 1–3 indicating alternative nutrition such as tube feeding, scores of 4–6 indicating a texture-modified diet, and a score of 7 indicating no restrictions on oral intake. In this study, each FOIS level corresponded to a specific food texture. For example, FOIS 4 indicated a pureed diet while FOIS 5 corresponded to a chopped diet. The FOIS assessment was conducted by nurses and speech-language pathologists twice, at admission and at discharge.

### Experimental variables

The living setting before hospitalisation (home or nursing faculty) was recorded from the information provided at admission. Home settings included individuals living independently or with family, often receiving home-visit care. Nursing facilities provided continuous medical and nursing care, reflecting differences in health conditions and caregiving.

### Covariates

Covariate data were recorded at admission, except for recurrent pneumonia, which was assessed during hospitalisation. Pneumonia severity was assessed using the pneumonia severity index (PSI). The total PSI score was calculated from the scores for patient background (age, sex, and nursing home resident), comorbid illnesses (neoplastic disease, liver disease, congestive heart failure, cerebrovascular disease, and renal disease), physical examination findings (altered mental status, respiratory rate ≥ 30/min, systolic blood pressure < 90 mmHg, temperature < 35 °C or ≥ 40 °C, and pulse ≥ 125/min), laboratory and radiographic findings (arterial pH < 7.35, blood urea nitrogen level ≥ 30 mg/dL, sodium level < 130 mEq/dL, glucose level ≧ 250 mg/dL, haematocrit < 30%, partial pressure of arterial oxygen < 90%, and pleural effusion) [18, 19]. The PSI severity was assessed based on a score categorising patients into three severity classes [20]. Patient frailty was assessed using the clinical frailty scale (CFS [21]). A cut-off value of 7 for the CFS score was selected based on a prior report that showed a higher 1-year mortality rate after admission among patients with a CFS score of ≥ 7 [22].

The disease history, including the history of aspiration pneumonia, was obtained from the medical records. Recurrent pneumonia during hospitalisation was defined as cases in which clinical pneumonia symptoms (such as fever ≥ 37.5 °C and increased sputum) returned after initial remission with antibiotic treatment during the observation period and antimicrobial therapy was re-infused at the discretion of the physician.

### Statistical analyses

Patient parameters are presented as mean ± standard deviation. The patients were classified into two groups according to whether they resided at home or in a nursing faculty immediately before disease onset. The normality of each variable was assessed for the entire sample and between groups using the Kolmogorov–Smirnov test, followed by appropriate statistical tests to compare differences.

Descriptive statistics of the FOIS score during hospitalisation were calculated, and the patients were divided into three groups: the group with alternative nutrition (FOIS score: 1–3), the group with texture-modified diet (FOIS score: 4–6), and the group with no oral intake restriction (FOIS score: 7). In addition, descriptive statistics of changes in the FOIS score during hospitalisation were calculated in the two groups according to the pre-hospital living setting.

To assess the effect of the pre-hospital living setting on the nutritional intake route upon discharge, a binary logistic regression analysis was conducted. The primary outcome was the nutritional intake route upon discharge, categorised as tube feeding (FOIS ≤ 3) or oral intake (FOIS ≥ 4). The secondary outcome was the presence of dysphagia at admission, which was categorised based on a FOIS score of 7 or below. Patients with a FOIS score of 6 or lower, who required texture-modified diets or tube feeding, were classified as having dysphagia. For the statistical analysis, the categorical dependent variables were defined as follows: dysphagia at admission was coded as 1 for presence and 0 for absence, and the nutritional intake route upon discharge was coded as 1 for tube feeding and 0 for oral intake.

The explanatory variables included pre-hospital living setting, age, sex, PSI score, CFS score, history of aspiration pneumonia, and recurrent pneumonia during hospitalisation. The explanatory variables were entered into the model using forced entry. The following dichotomized variables were included in the model: pre-hospital living setting (home = 0, nursing facility = 1), sex (male = 0, female = 1), CFS score (< 7 = 0, ≥ 7 = 1), history of aspiration pneumonia (no = 0, yes = 1), and recurrent pneumonia during hospitalisation (no = 0, yes = 1). All the statistical analyses were performed using SPSS version 25 (IBM Inc., Tokyo, Japan).

### Ethical considerations

This study was approved by the Institutional Ethics Committee of Juntendo Tokyo Koto Geriatric Medical Centre (approval no. 111 − 10) and conducted in accordance with the Declaration of Helsinki. Sufficient explanation was provided to the patient in writing and verbally, and written informed consent was obtained from the patient or their family by one of five physicians [23]. This study used the same dataset as that in previous studies; however, the previous studies did not examine the effect of the pre-hospital living setting on oral intake at discharge [16, 23].

## Results

This study included 106 participants; of these, 16 were excluded due to missing data and one was excluded for being under 65 years old, resulting in 89 participants for analysis. During the hospital stay, 14 participants passed away, and 75 were discharged. The groups of patients admitted from home and from a nursing facility included 67 (75.2%) and 22 (24.7%) patients, respectively (Table 1). Age was normally distributed in the entire sample. In the group-specific analysis, both age and PSI also followed a normal distribution, while the other measured variables did not. The patients admitted from a nursing facility had a higher PSI score (*p* < 0.001) and worse condition, such as altered mental status (*p* = 0.031), respiratory rate ≥ 30 breaths/min (*p* = 0.049), and dementia (*p* = 0.009), than those admitted from their home (Table 1). No patients met the criteria for body temperature < 35 °C (95 °F) or > 39.9 °C (103.8 °F) as part of the PSI severity assessment. Additionally, the nursing facility group had a significantly higher prevalence of dysphagia at admission (*p* = 0.029) and tube feeding upon discharge (*p* = 0.001) (Table 1).

**Table 1:** Patient characteristics (*n* = 89)

	Home group (*n* = 67)	Nursing facility group (*n* = 22)	*P*-value	Total (*n* = 89)
Age, years	84.2 ± 6.8	86.6 ± 4.9	0.221^§^	84.8 ± 7.9
Sex (men), n (%)	41 (61.2)	11 (50.0)	0.355^‡^	52 (58.4)
CFS score ≥ 7, n (%)	38 (56.7)	16 (72.7)	0.180^‡^	54 (60.7)
PSI, score	113.9 ± 33.5	144.3 ± 43.2	< 0.001^§,†^	121.4 ± 38.2
PSI			0.002^*,‡^	
Low risk, n	16	2		18
Moderate risk, n	35	5		40
High risk, n	16	15		31
Presence of dysphagia at admission (FOIS score ≤ 6), n	45	20	0.029^*,‡^	65
Neoplastic disease, n (%)	17 (25.4)	5 (22.7)	0.803^‡^	22 (24.7)
Cerebrovascular disease history, n (%)	17 (25.4)	3 (13.6)	0.252^‡^	20 (22.5)
Liver disease history, n (%)	1 (1.5)	2 (9.1)	0.087^‡^	3 (3.4)
CHF history, n (%)	10 (14.9)	4 (18.2)	0.972^‡^	14 (15.7)
Renal disease history, n (%)	4 (9.5)	1 (8.3)	0.801^‡^	5 (5.6)
Altered mental status, n (%)	17 (25.4)	11 (50.0)	0.031^*,‡^	28 (31.5)
Respiratory rate ≥ 30 breaths/min, n (%)	5 (7.5)	5 (22.7)	0.049^*,‡^	10 (11.2)
Systolic blood pressure < 90 mmHg, n (%)	3 (4.5)	3 (13.6)	0.137^‡^	6 (6.7)
Pulse ≥ 125 beats/min, n (%)	3 (4.5)	0 (0.0)	0.313^‡^	3 (3.4)
pH < 7.35, n (%)	2 (3.0)	2 (9.1)	0.230^‡^	4 (4.5)
BUN level ≥ 30 mg/dL or ≥ 11 mmol/L, n (%)	19 (28.4)	7 (31.8)	0.757^‡^	26 (29.2)
Sodium level < 130 mmol/L, n (%)	3 (4.5)	3 (13.6)	0.137^‡^	6 (6.7)
Glucose level ≥ 250 mg/dL or ≥ 14 mmol/L, n (%)	8 (11.9)	6 (27.2)	0.087^‡^	14 (15.7)
Haematocrit < 30%, n (%)	5 (7.5)	2 (9.1)	0.806^‡^	7 (7.9)
Partial pressure of oxygen < 60 mmHg or < 8 kPa, n (%)	23 (34.3)	11 (50.0)	0.189^‡^	34 (38.2)
Pleural effusion on a radiograph, n (%)	13 (19.4)	6 (27.3)	0.434^‡^	19 (21.3)
Parkinson’s disease, n (%)	8 (11.9)	2 (9.1)	0.713^‡^	10 (12.0)
History of aspiration pneumonia, n (%)	16 (23.9)	5 (22.7)	0.912^‡^	21 (23.6)
Diabetes mellitus, n (%)	12 (17.9)	2 (9.1)	0.324^‡^	14 (16.9)
Dementia, n (%)	45 (67.2)	21 (95.5)	0.009^*,‡^	66 (74.2)
Length of hospital stay (*n* = 75), days	42.3 ± 36.9	48.8 ± 35.7	0.388^†^	44.0 ± 36.566
Nutritional intake route upon discharge			0.001^*,‡^	
Oral intake, n (%)	39 (86.7%)	6 (13.3%)		45 (60.0%)
Alternative nutrition, n (%)	16 (53.3%)	14 (46.7%)		30 (40.0%)
Death (*n* = 89), n (%)	12 (17.9)	2 (9.1)	0.324^‡^	14 (16.9)

*Abbreviations* BUN, blood urea nitrogen; CFS, clinical frailty scale; CHF, congestive heart failure; FOIS, functional oral intake scale; PSI, pneumonia severity index

^*^Statistically significant (*P* < 0.05)

^§^ t-test

^†^Mann–Whitney test

^‡^χ^2^ test

At admission, 24 patients (27.0%) were on a regular diet. By discharge, this decreased to 7 patients (9.3%), and alternative nutrition increased from 5 (5.6%) to 30 patients (40.0%). In patients from nursing facilities, alternative nutrition use rose sharply from 2 (9.1%) to 14 patients (70.0%) (Table 2).

**Table 2:** Change in FOIS score during hospitalisation

	Total		Home group		Nursing facility group	
	At admission (*n* = 89)	At discharge (*n* = 75)	At admission (*n* = 67)	At discharge (*n* = 55)	At admission (*n* = 22)	At discharge (*n* = 20)
No oral intake restriction (FOIS score = 7)	27.0	9.3	32.8	12.7	9.1	0.0
Texture-modified diet (4 ≦ FOIS score ≦ 6)	67.4	50.7	62.7	58.2	81.8	30.0
Alternative nutrition (1 ≦ FOIS score ≦ 3)	5.6	40.0	4.5	29.1	9.1	70.0

Values are presented as percentages

*Abbreviations* FOIS, functional oral intake scale

A binary logistic regression analysis with the presence of dysphagia at admission as the dependent variable found that the CFS (odds ratio (OR) = 12.63, 95%CI = 3.41–46.80, *p* < 0.001) was an independent explanatory variable (Table 3).

**Table 3:** Association between pre-hospital living setting and presence of dysphagia at admission (*n* = 89)

		Univariate		Multivariate	
Dependent variable	Independent variable	OR (95%CI)	*P*- value	OR (95%CI)	*P*- value
Presence of dysphagia at admission (*n* = 89)	Pre-hospital living setting	4.889 (1.048–22.813)	0.043^*^	4.010 (0.660–24.356)	0.131
	Age	1.001 (0.943–1.062)	0.979	0.987 (0.918–1.061)	0.716
	Sex	0.788 (0.3.7–2.025)	0.621	0.448 (0.120–1.676)	0.233
	PSI score	1.019 (1.003–1.036)	0.021^*^	1.014 (0.992–1.038)	0.218
	CFS score	8.471 (2.886–24.865)	< 0.001^*^	12.627 (3.407–46.802)	< 0.001^*^
	History of aspiration pneumonia	4.543 (0.971–21.258)	0.055	5.392 (0.920–31.589)	0.061

*Abbreviations* OR, odds ratio; CI, confidential interval; FOIS, functional oral intake scale; PSI, pneumonia severity index; CFS, clinical frailty scale. ^*^Statistically significant (*P* < 0.05)

A binary logistic regression analysis with the nutritional intake route upon discharge as the dependent variable revealed that the pre-hospital living setting (OR = 7.72, 95%CI = 1.67–35.10, *p* = 0.008), age (OR = 0.90, 95%CI = 0.82–0.99, *p* = 0.022), CFS (OR = 5.92, 95%CI = 1.30–27.03, *p* = 0.022), and recurrence of pneumonia during hospitalisation (OR = 7.10, 95%CI = 1.85–27.20, *p* = 0.004) were independent explanatory variables (Table 4).

**Table 4:** Association between pre-hospital living setting and nutritional intake route upon discharge (*n* = 75)

		Univariate		Multivariate	
Dependent variable	Independent variable	OR (95%CI)	*P*- value	OR (95%CI)	*P*- value
Nutritional intake route upon discharge (*n* = 75)	Pre-hospital living setting	5.687 (1.857–17.422)	0.002^*^	7.716 (1.696–35.095)	0.008^*^
	Age	0.973 (0.918–1.031)	0.352	0.901 (0.823–0.985)	0.022^*^
	Sex	1.368 (0.541–3.463)	0.508	1.789 (0.506–6.319)	0.366
	PSI score	1.011 (0.998–1.024)	0.095	1.004 (0.989–1.020)	0.599
	CFS score	4.375 (1.421–13.472)	0.010^*^	5.920 (1.297–27.029)	0.022^*^
	Recurrence of pneumonia during hospitalisation	4.571 (1.642–12.725)	0.004^*^	7.098 (1.852–27.200)	0.004^*^

*Abbreviations* OR, odds ratio; CI, confidential interval; FOIS, functional oral intake scale; PSI, pneumonia severity index; CFS, clinical frailty scale. ^*^Statistically significant (*P* < 0.05)

## Discussion

In this study, 75.2% and 24.7% of the participants were included in the home and nursing facility groups, respectively. The pre-hospital living setting was associated with the nutritional intake route upon discharge. CFS was associated with both dysphagia at admission and the nutritional intake route upon discharge. In addition, a higher proportion of patients admitted from nursing facilities had higher CFS scores.

This study demonstrated that the pre-hospital living setting is associated with the nutritional intake route upon discharge, even after adjusting for the confounding factors. Specifically, patients admitted from nursing facilities had 7.72 times higher odds (95% CI: 1.67–35.10, *p* = 0.008) of requiring alternative nutrition upon discharge compared to those admitted from home. This highlights the strong association between pre-hospital living setting and nutritional intake route at discharge. In this study, patients admitted from nursing facilities had poorer consciousness levels, a higher proportion of dementia, and worse PSI scores than patients admitted from home (Table 1). Generally, patients admitted from nursing facilities were in poorer condition than those admitted from home. This, combined with the influence of unadjusted confounding factors, might have made pre-hospital living setting an independent factor associated with nutritional intake route upon discharge. In addition, a total of 54 patients (60.7%) in this study had a CFS score greater than 7, indicating their need for care. Older people who need care have weak host resistance due to multiple diseases and multiple medications [24]. Exacerbation of aspiration pneumonia occurs when the burden of aspirated material significantly overwhelms the body’s natural defences. Frailty is one of the poor prognostic factors in patients with pneumonia, as frail older adults have reduced resilience, and the concept of frailty-associated pneumonia has been proposed [12].

The guidelines from the Japanese Respiratory Society define NHCAP independently of CAP and HAP [25], with previous research on the vulnerability of nursing facility residents supporting the results of this study. Nursing facilities in Japan are available in both private and public formats. The services they offer and their costs vary widely, as they cater to a diverse range of individuals, from independent older adults to those requiring advanced care. Furthermore, resident outcomes in facility care vary significantly based on the quality of the caregiving staff [26]. In contrast, home care is typically provided through collaboration between family members and relatively stable helpers, making differences due to staff variability less likely than that in nursing facilities. Moreover, the primary goal of acute care hospitals is the treatment of pneumonia, not the rehabilitation of dysphagia. Unless the patient is recovering from acute medical conditions such as stroke, where natural recovery of swallowing function is more common, significant improvements in swallowing function are unlikely. Given that aspiration pneumonia often recurs between hospital and community settings, it is plausible that both the patient’s frailty and pre-hospital factors are key predictors of oral intake ability at discharge.

Recurrent pneumonia during hospitalisation was an independent factor associated with nutritional intake route upon discharge. In this study, the recurrence rate (39.3%) was higher than that in a previous study [27], which may be because the patients in this study were older than those in the previous study (80 vs. 85 years). Many older patients with aspiration pneumonia in this study experienced a significant decrease in their FOIS score during hospitalisation, and from admission to discharge, the proportion of patients receiving alternative nutrition increased from 5.6 to 37.3%. The trend was especially pronounced among patients admitted from facilities, for whom the proportion of patients receiving alternative nutrition rose sharply from 9.1 to 70%. In a retrospective cohort study of 28 Canadian hospitals targeting patients admitted with aspiration, approximately 7% were tube-fed. Although the resulting percentage was different, the participants in this study were patients with aspiration pneumonia, who were older and likely were in poorer general condition with higher CFS [28]. Diet texture and swallowing function vary among community-dwelling individuals [8], and a mismatch between diet texture and masticatory and swallowing functions causes adverse events, such as aspiration and choking [29, 30]. There is a concern that patients with aspiration pneumonia are quickly deemed unfit for oral intake without adequate evaluation of their swallowing ability in the acute stage [31]. Dysphagia is an independent factor associated with unplanned hospitalisation in institutionalised older patients [32]. Continuous evaluation of swallowing function and oral health status by healthcare professionals, including speech-language pathologists and nurses, is also important in the community setting after discharge from the hospital [33, 34]. Furthermore, implementing systematic programmes within facilities to screen for and manage residents with dysphagia to the greatest extent possible could be effective in preventing hospital admissions of frail residents [35, 36]. For example, establishing a safe swallowing culture within the facility through dysphagia education and support by speech-language pathologists can contribute to this goal [36]. If a community lacks medical resources for managing aspiration pneumonia and related dysphagia, optimising medical allocation using telemedicine should be considered [37, 38].

This study has certain limitations. First, this study had a small sample size, as it was based on the number of patients hospitalised during a defined period rather than an a priori sample size calculation. This limitation may introduce bias or reduce statistical power, potentially affecting the reliability of the results. Future studies with larger sample sizes and proper sample size calculations are warranted to minimise potential errors and confirm these findings. We recorded whether the pre-hospital living setting was a home or a nursing facility but did not collect more detailed information such as facility type (private or public) or staffing levels. Unaccounted differences in socio-economic, educational, cultural, and family support between these settings may have introduced bias. Although we adjusted for some confounders, unmeasured environmental factors may still have influenced the outcomes. Future studies should incorporate more detailed information about pre-hospital environments to better understand their impact on patient care and outcomes. It should be noted that a decline in the FOIS score does not necessarily indicate a deterioration in function, but may also reflect appropriate dietary management to address the mismatch between functional capacity and food consistency.

Despite these limitations, this study provides some important clinical findings. The pre-hospital living setting, which is a very simple and fundamental piece of information, is a useful factor in predicting the FOIS score during hospitalisation. Since the nutritional pathway at discharge also affects the subsequent prognosis, medical staff responsible for older patients with aspiration pneumonia should pay attention not only to physical information but also to the environmental factors of the patient’s pre-hospital living setting. To prevent a significant decrease in the FOIS score, which greatly impairs the patient’s quality of life [39, 40], it is extremely important for hospital medical staff to avoid HAP.

In conclusion, differences in pre-hospital living setting are significantly associated with nutritional intake route upon discharge. Specifically, patients admitted from nursing facilities had 7.72 times higher odds of requiring alternative nutrition upon discharge compared to those admitted from home. CFS is significantly associated with oral intake both at admission and discharge, and pneumonia recurrence during hospitalisation contributes to the decline in the FOIS score. For older adults with high levels of frailty, in particular, it is important to implement screening and management of dysphagia, especially in nursing facilities, and to focus on preventing pneumonia recurrence when they are hospitalised. Therefore, it is essential to continuously address swallowing disorders across both facility and hospital settings to ensure comprehensive care.

## Data Availability

The datasets used for this study are available from the corresponding author on reasonable request.

## Abbreviations

BUN: Blood urea nitrogen
CAP: Community-acquired pneumonia
HAP: Hospital-acquired pneumonia
CFS: Clinical frailty scale
CHF: Congestive heart failure
CI: Confidential interval
FOIS: Functional oral intake scale
NHCAP: Nursing and healthcare-associated pneumonia
OR: Odds ratio
PSI: Pneumonia severity index
